# Mechanical Properties and Anti-Spalling Behavior of Ultra-High Performance Concrete with Recycled and Industrial Steel Fibers

**DOI:** 10.3390/ma12050783

**Published:** 2019-03-07

**Authors:** Juan Yang, Gai-Fei Peng, Guo-Shuang Shui, Gui Zhang

**Affiliations:** Faculty of Civil Engineering, Beijing Jiaotong University, Beijing 100044, China; gfpeng@bjtu.edu.cn (G.-F.P.); gsshui@bjtu.edu.cn (G.-S.S.); guizhang@bjtu.edu.cn (G.Z.)

**Keywords:** ultra-high performance concrete, recycled steel fiber, industrial steel fiber, mechanical properties, explosive spalling

## Abstract

Experimental investigations on the mechanical properties of ultra-high performance concrete (UHPC) incorporating two types of recycled steel fiber processed from waste tires and three types of industrial steel fiber were carried out for comparison. Mechanical properties of UHPC include compressive strength, splitting tensile strength, fracture energy, and elastic modulus. Their explosive spalling behaviors under high temperatures were also investigated. The results show that all types of steel fiber exhibit a beneficial effect on the mechanical properties and the anti-spalling behavior of UHPC, except that recycled steel fiber with rubber attached (RSFR) has a slightly negative effect on the compressive strength of UHPC. Compared to industrial steel fibers, recycled steel fibers have a more significant influence on improving the splitting tensile strength and fracture energy of UHPC, and the improvement of RSFR was much higher than that of recycled steel fiber without rubber (RSF). UHPC that incorporates industrial hooked-end steel fiber (35 mm in length and 0.55 mm in diameter) exhibits the best resistance to explosive spalling, and the second is the RSF reinforced UHPC. The positive relationship between the fracture energy and the anti-spalling behavior of steel fiber reinforced UHPC can be presented. These results suggest that recycled steel fiber can be a toughening material and substitute for industrial steel fibers to be used in ultra-high performance concrete, especially RSFR.

## 1. Introduction

Environmental concerns associated with waste rubber tires have attracted significant attention in recent years. A number of related associations and councils have been established in numerous countries, such as the Tire Industry Association and the Rubber Division of the American Chemical Society. Additionally, because waste rubber tires are not biodegradable, the disposal of waste tires in landfills has been banned by law [1]. 

In view of environmental protection and economic benefit, more and more attention has been paid to the recycling of waste rubber tires. Recycling of waste rubber tires has focused on extracting the rubbers and steel fibers. A large number of studies have reported the applications of these materials in many types of concretes [2,3,4,5,6,7,8,9]. It has also been confirmed that the mechanical properties of concrete reinforced with recycled steel fiber from tires are comparable to those of concrete with industrial steel fibers [10]. The thickness of concrete pavement could be reduced by up to 26% due to the addition of recycled steel fibers when only taking into account their effect on the fatigue property [11]. An economic analysis of recycled steel fiber reinforced concrete found that using recycled steel fiber alone could save up to 33% [12]. Moreover, a comprehensive study was conducted to evaluate the anchoring characteristics of recycled steel fiber through a pull-out test, the flexural capacity of steel fiber reinforced concrete, and new stress–strain models for design purposes [13]. Another result revealed that the combination of silica fume and recycled steel fiber improved the mechanical properties and impact resistance of specimens [14]. However, few of the results on the application of recycled steel fibers in ultra-high performance concrete (UHPC) can be found.

Ultra-high performance concrete (UHPC) has excellent strength performance and super durability. The excellent properties have led to increasing use of UHPC in engineering structures, such as ultra-high-rise buildings in Japan [15,16], military shielding panels in Germany [17], the National Great Theater [18], the International Finance Center [19], and Huangchao Wanxin Building in China [20]. Applications have focused on optimizing its use by reducing concrete member thickness [21], reducing the size of anchorage blocks and amounts of reinforcements in the pre-stressed concrete structures [22], securing economic efficiency by achieving a lightweight superstructure [23], and realizing sufficient connection performance of lap-spliced cast-in-place joints in bridge deck slabs [24]. Under impact loading, UHPC was approximately twice as strong as conventional fiber reinforced concrete and exhibited excellent impact resistance [25]. UHPC can be used for the reinforcement of structure elements but developing the applicable confinement model is needed [26]. Additionally, many existing bridges built many years ago have exhibited poor quality, such as low shear capacity, the inappropriate location of lap splices in pier members, and a lack of adequate reinforcement details [27,28], and there is an urgent need to further assess their performance and then strengthen them [29,30]. Adding UHPC layers to the existing components can upgrade these conventional reinforced concrete structures [31]. 

However, because of the lower water to binder ratio (W/B), the microstructure of UHPC incorporating three types of mineral admixtures is much denser. Results indicated that the tensile strengths of concrete specimens decreased with the increasing addition of mineral admixtures [32]. The axial tensile properties of concrete are important parameters which have great effects on crack resistance, and the denser microstructure of concrete also results in the poor cracking resistance. Therefore, UHPC without any steel fiber exhibits high brittleness and is more prone to encounter explosive spalling when exposed to high temperatures [33]. Adding steel fiber is a common approach for improving the toughness of UHPC due to its crack resistance, resulting in the significant improvement of the shear capacity and bonding performance of UHPC. It has been shown that increasing the dosage of steel fiber in UHPC beams without shear stirrups did not only increase the shear capacity of the beams but also changed their failure modes from shear tension to shear compression [34,35]. Specifically, the use of steel fibers as a better alternative to shear reinforcement in high-strength concrete beams can be considered [36]. Steel fiber improves the bonding performance of concrete elements with lap-spliced reinforced bars remarkably, suggesting that the UHSC structure increase the volume fraction of steel fibers rather than increase splice length to ensure bonding strength [37]. Moreover, the fiber reinforcement can significantly change the shear behavior and increase the shear capacity [38], even in the fiber-reinforced UHPC without shear reinforcement [39]. Additionally, the steel fiber reinforced cementitious composite (SFRCC) jacketing can be an appropriate alternative to the carbon fiber reinforced polymers external reinforcement for the reinforcement of existing poor-quality concrete structures [40]. An innovative application of SFRCC for the repair and retrofit of damaged columns is also proposed and validated experimentally [41]. In terms of ductility, concrete specimens strengthened with the SFRCC did not show a sensitive increase of deformation capacity in the post-peak phase during loading [42]. These conclusions show that there are many advantages in the design practice of the steel fiber reinforced UHPC. 

Explosive spalling of concrete under high temperatures is, in essence, severe cracking, which steel fibers also have a positive effect on improving the resistance to [43,44]. However, influences of steel fiber on preventing concrete from explosive spalling still remain controversial, not only in high-strength or high-performance concrete [45,46] but also in reactive powder concrete [47,48]. Additionally, a researcher proposed that the positive effect of steel fiber on improving the anti-spalling behavior of high and ultra-high performance concrete can be ignored [49]. 

Based on the above mention, further investigations on the mechanical properties and explosive spalling behavior of UHPC that incorporates different types of steel fiber are needed. In this investigation, compressive strength, splitting tensile strength, fracture energy, elastic modulus, and explosive spalling behavior of plain UHPC and UHPC reinforced with two types of recycled steel fiber and three types of industrial steel fiber were determined for comparison to explore the characteristics of steel fiber reinforced UHPC and find out the optimum steel fiber for the optimum UHPC.

## 2. Materials and Methods 

### 2.1. Materials

**Figure 1:**
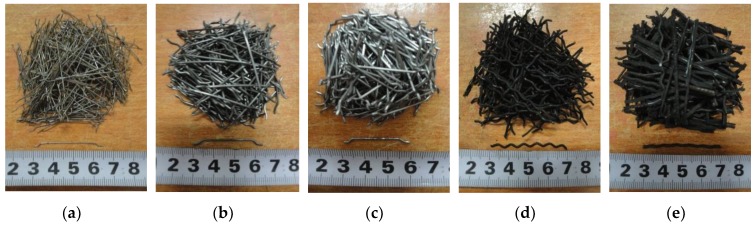
Steel fibers in this investigation: (**a**) Steel fiber (SF)1; (**b**) SF2; (**c**) SF3; (**d**) recycled steel fiber (RSF); (**e**) recycled steel fiber with rubber (RSFR).

Concretes were prepared using ordinary raw materials. 52.5R Portland cement and silica fume (SF) were used as binders. Basalt with two particle sizes in the ranges of 5–10 mm and 10–16 mm by a mass ratio of 3:7 was used as the coarse aggregate. Artificial sand was used as fine aggregate. Five different types of steel fibers are shown in Figure 1 and Table 1. Polycarboxylate superplasticizer with a solid content of 50% was used to maintain excellent workability of fresh concrete. 

### 2.2. Concrete Preparation 

Six types of UHPC specimens prepared in the forms of 100 × 100 × 100 mm cube and 100 × 100 × 300 mm beam were designated as Plain/UHPC, SF1/UHPC, SF2/UHPC, SF3/UHPC, RSF/UHPC, and RSFR/UHPC. Their mix proportions are given in Table 2. After casting, each specimen was immediately wrapped in plastic to minimize moisture loss and stored at room temperature for 24 hours. Next, these specimens were de-molded and placed in a tank of water at a constant temperature of 20 °C for 56 days.

### 2.3. Test Methods

#### 2.3.1. Concrete Strength and Static Modulus of Elasticity 

Strength tests and static elastic modulus tests on all types of UHPCs at the age of 28 days were performed according to a Chinese standard GB/T 50081-2002 Standard for test method of mechanical properties on ordinary concrete [50]. The data presented in this study are the average value of the three specimens tested. 

Cube specimens of 100 mm × 100 mm × 100 mm were employed for the compressive strength test. The specimens were loaded using a testing machine of 200-ton capacity at the rate of 1.0 MPa/s until failure. Cube specimens of 100 mm size were used to determine the splitting tensile strength at a loading rate of 0.1 MPa/s. 

Six prismatic specimens of 100 mm × 100 mm × 300 mm were employed to determine the static modulus of UHPC. Firstly, three specimens were used to determine their axial compressive strength and their average strength value obtained was the result. Secondly, two deformation measuring instruments were mounted on the center line on both sides of the specimen symmetrically, and then the center position of the specimen was adjusted repeatedly to make the difference less than 20% between the deformation values and their average value. Thirdly, during loading, the load values and deformation values were recorded. Finally, the static modulus of elasticity of concrete could be calculated by using Equation (1)

*E*_c_ = [(*F*_a_ − *F*_0_)/A] × [*L*/(*ε*_a_ − *ε*_0_)] (1)

where *E*_c_ is the static modulus of elasticity (MPa); *F*_a_ is the load when the stress is 1/3 of the axial compressive strength (N); *F*_0_ is the initial load when the stress is 0.5 MPa; A is the pressure area of specimen (mm^2^); *L* is the measuring scale of 100 mm; *ε*_a_ is the average deformation value of both sides when load is *F*_a_ (mm); *ε*_0_ is the average deformation value on both sides when load is *F*_0_ (mm).

#### 2.3.2. Fracture Energy Test

**Figure 2:**
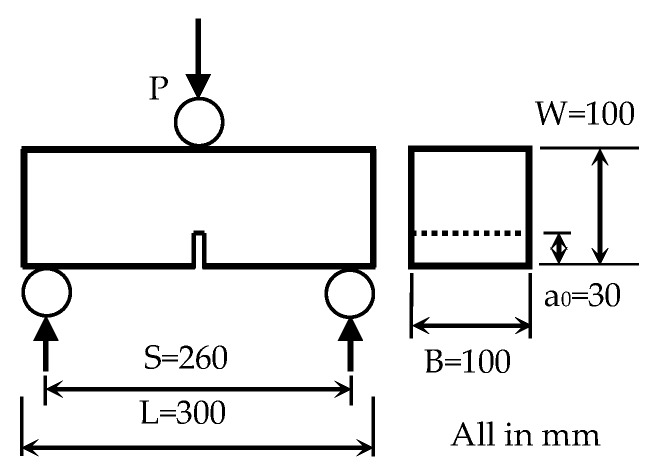
Configuration of three-point bending notched beam specimen.

Notched beam specimens of 100 × 100 × 300 mm were employed to determine the fracture energy at the age of 56 days. The fracture energy was determined according to a RILEM test method [51]. The configuration of the tested specimen is shown in Figure 2. A notch was prepared during the casting of specimens to form a crack at the mid-span of each specimen. In this investigation, the notch depth was 30 mm. 

A three-point bending test was conducted on a notched beam specimen. The loading was displacement controled at a rate of 0.05 mm/min. The mid-span deflection δ was recorded during the whole loading process until failure. From the recorded load–deflection curve, the fracture energy of concrete could be calculated by using Equation (2) as specified in the RILEM test method [51].

(2) $$G_{F}=\left[\int_{0}^{\delta_{0}}P\left(\delta \right)+mg\delta_{0}\right]/A_{lig}$$

where $G_{F}$ is the fracture energy (J/m^2^), $m=m_{1}+m_{2}$ (kg), $m_{1}=Ms/L$ (the weight of the beam support, calculated as beam weight multiplied by s/L), M is the mass of the specimen, $m_{2}$ is the weight of the part of the loading arrangement not attached to the machine but that follows the beam until failure, s is the span, L is the length of the specimen, g=9.81 m/s^2^, $\delta_{0}$ is the mid-span deflection of the specimen at failure (m), $A_{lig}$ is the area of the ligament (m^2^), δ is the mid-span deflection (m), and P is the load (N).

#### 2.3.3. Explosive Spalling Test

**Figure 3:**
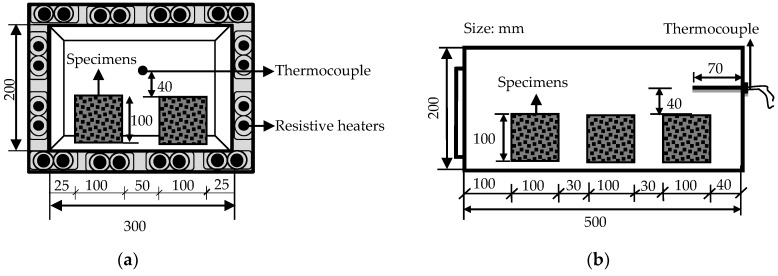
Heating setup for the explosive spalling test. (**a**) Front view; (**b**) lateral view.

Explosive spalling tests were conducted on the 100 mm cube specimens after they were stored at room temperature for 365 days. The test method is according to the reported literature [52]. Specimens were heated in a muffle furnace from room temperature to 800 °C at a rate of 10 °C/min. The temperature was measured by a thermocouple located in the air. The thermocouple was positioned approximately 40 mm above the forming surface of the concrete specimen inside the electric furnace, as shown in Figure 3. 

## 3. Results

### 3.1. Mechanical Properties

**Figure 4:**
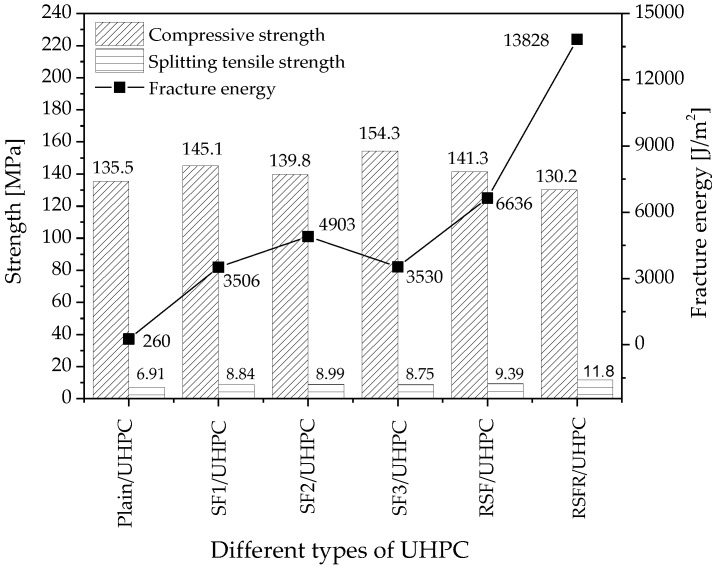
Compressive strengths, splitting tensile strengths, and fracture energies of UHPCs with various steel fibers and plain UHPC.

The compressive strength, splitting tensile strength, and fracture energy of UHPCs with different types of steel fiber and plain UHPC are shown in Figure 4. 

#### 3.1.1. Compressive Strength

The results of the influence of adding fibers on the compressive strength of mixtures are indicated in Figure 4. Adding steel fiber SF1, SF2, SF3, and RSF improved the compressive strength of plain UHPC by 7.1%, 3.2%, 13.9%, and 4.3%, respectively. The improvement can be attributed to the frictional stress and mechanical anchorage provided by the geometry of hooked-end industrial steel fibers and corrugated RSF [53]. The compressive strength of SF1/UHPC and SF2/UHPC revealed that steel fiber with a higher aspect ratio more effectively enhanced the compressive strength of UHPC than that with a lower aspect ratio. Similarly, steel fiber with an aspect ratio of 80 increased the compressive strength of concrete more efficiently than that with an aspect ratio of 40 [54]. Additionally, the effect of the aspect ratio of steel fiber on the compressive strength of concrete is related to its volume fraction. Concretes with a fiber volume of 1.5%, 1.0%, and 0.5% have the highest compressive strength with the aspect ratios of 45, 65, and 80, respectively [55].

The compressive strength of UHPC that incorporates industrial steel fiber SF3 with a tensile strength of 1800–2000 MPa was 10.4% higher than that reinforced by SF2 with the lower tensile strength of 900 MPa. However, the effect of the tensile strength of steel fiber itself on increasing the compressive strength of concrete was not significant and therefore may require more research [56]. 

Among all of the types of steel fibers, only RSFR had a slightly negative effect on the compressive strength of UHPC. The compressive strength of UHPC that incorporated RSFR was lower than that of plain UHPC by 5.3 MPa. This difference may be attributed to both the weak interfacial bonding between rubber particles and the cement matrix due to its hydrophobic nature and the stress concentration generated by rubber particles with much lower stiffness. Additionally, the decrease in compressive strength of RSFR/UHPC may be related to the increased air voids of mixtures due to the rubber-attached RSFR [57]. When the rubber in the RSFR was disposed of, the adverse effect of the steel fiber on the compressive strength of UHPC was eliminated, as demonstrated by the compressive strength of RSF/UHPC which was higher than that of plain UHPC.

#### 3.1.2. Splitting Tensile Strength 

Figure 4 indicates the efficiency of steel fibers on the splitting tensile strength of UHPC. All types of steel fibers—SF1, SF2, SF3, RSF, and RSFR—significantly improved the splitting tensile strength of plain UHPC by 27.9%, 30.1%, 26.6%, 35.9%, and 70.8%, respectively. The improvements of both recycled steel fibers were higher than industrial steel fiber, especially the RSFR. The efficient influence can be attributed to the high capacity of steel fibers in hindering the further crack propagation, resulting in the fiber bridging and the increase of the splitting tensile strength of concrete. 

The splitting strength of UHPC with industrial steel fibers SF1, SF2, and SF3 was 8.84 MPa, 8.99 MPa, and 8.75 MPa, respectively. It indicates that the effect of the aspect ratio and tensile strength of hooked-end steel fiber on the splitting tensile strength of UHPC were not pronounced. Similarly, a study showed that the splitting tensile strengths of concretes with the aspect ratios of 45, 60, and 80 were 4.50 MPa, 4.51 MPa, and 4.58 MPa, respectively [55]. However, the tensile strength of steel fiber had a significant influence on the splitting tensile strength of concrete [56]. The splitting tensile strength of UHPC reinforced with RSF was slightly higher than that of UHPC with industrial steel fibers. This result may be attributed to the fact that RSF is 5 mm longer and has a corrugated shape. A study reported that the carbon black on the surface of RSF decreased the flexural strength of concrete by approximately 15% compared to concrete that incorporated industrial steel fibers with a tensile strength similar to that of RSF [13]. The effect of carbon black on mechanical properties and pore microstructure of UHPC needs further research. 

Additionally, the influence of recycled steel fiber RSFR in improving the splitting tensile strength of UHPC was superior to that of RSF, which was attributed to the combination of the beneficial effect of the synergistic action between the rubber-attached RSFR and steel fiber itself, as well as the disadvantageous effect resulting from high-temperature damage to RSF during heat treating. 

#### 3.1.3. Fracture Energy 

The fracture energies of UHPC that incorporate different types of steel fiber are shown in Figure 4. Steel fiber improved the fracture energy of plain UHPC significantly. Recycled steel fibers exhibited the best efficiency. The fracture energies of RSFR/UHPC and RSF/UHPC were 13,828 J/m^2^ and 6636 J/m^2^, respectively, while those of the UHPCs reinforced with industrial steel fibers were less than 5000 J/m2. Therefore, the capacity of UHPC to absorb energy is greatly improved by recycled steel fibers, especially RSFR. Many previous studies also identified the capacity of recycled waste tires to absorb fracture energy [58]. Therefore, its excellent ability to improve the fracture toughness of UHPC suggests that recycled steel fiber has a promising application in concrete. 

Except for the fracture energy value in Figure 4, the other data obtained also demonstrate that RSFR can remarkably improve the fracture energy of UHPC. 

**Figure 5:**
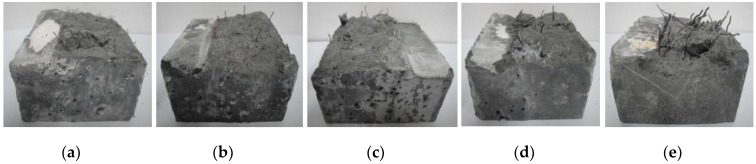
Morphology of steel fibers on the fracture surfaces of specimens after fracture energy testing. (**a**) SF1; (**b**) SF2; (**c**) SF3; (**d**) RSF; (**e**) RSFR.

Firstly, the morphology of pulled-out steel fibers on the fracture surfaces of the notched specimens after fracture energy testing indicates the differences among the exposed sections of steel fibers, as shown in Figure 5. This illustrates that the exposed section of RSFR is the longest among all of the steel fibers. The longer the length was, the more time the fracture process took to absorb energy and the higher the fracture energy of UHPC was.

**Figure 6:**
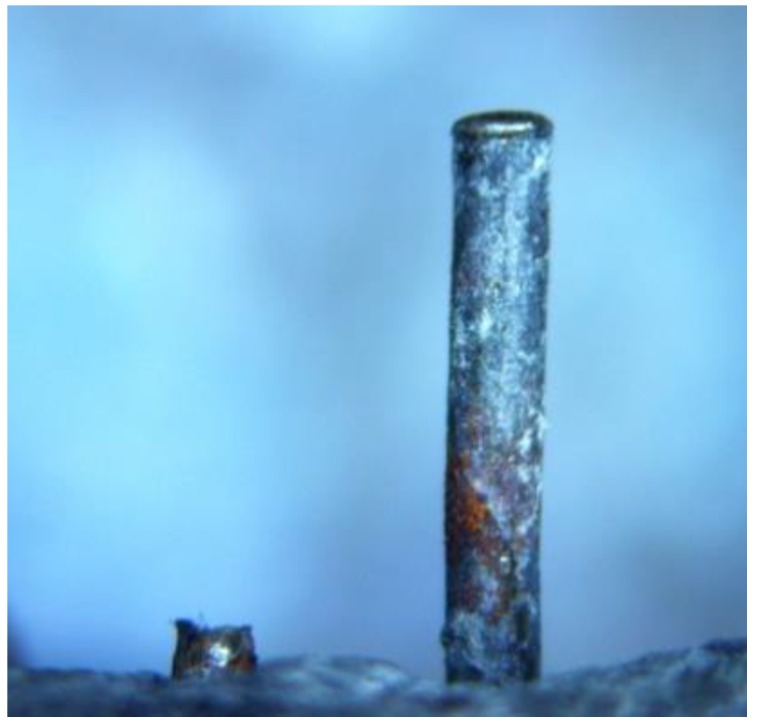
Ruptured and pulled-out steel fibers on a fracture surface observed by an optical microscope after fracture energy testing.

Secondly, steel fiber was ruptured or pulled out during fracture energy testing. The appearance of a ruptured steel fiber and a pulled-out fiber on the fracture surface was observed by an optical microscope, as is shown in Figure 6. The data in Table 3 provide an accurate number of ruptured or pulled-out fibers on the fracture surfaces of the specimens. There were more pulled-out fibers than ruptured fibers for recycled steel fibers and SF3. For SF1 and SF2, there were more ruptured steel fibers. Pulled-out steel fiber with a long exposed section absorbed more energy than ruptured fibers, resulting in the high fracture energy of concrete.

**Figure 7:**
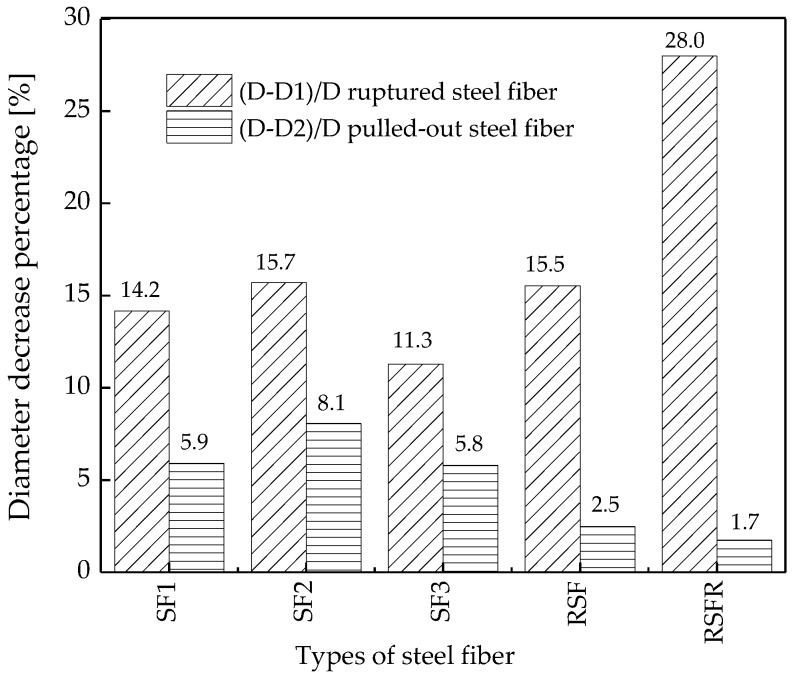
Percentage decrease of the diameter of steel fibers during fracture energy testing.

Thirdly, as shown in Figure 6, D1 and D2 were used to label the end diameters of the ruptured steel fiber and pulled-out steel fiber, respectively. The average value of ten steel fibers demonstrated that the end diameters of all ruptured steel fibers or pulled-out steel fibers were smaller than their natural diameters (D). The decreased percentage of the end diameters is presented in Figure 7. The decreased percentage of the end diameter of the ruptured RSFR was much higher than that of other steel fibers. Therefore, RSFR underwent the largest tensile deformation during fracture energy testing and absorbed the most energy among all types of steel fiber.

**Figure 8:**
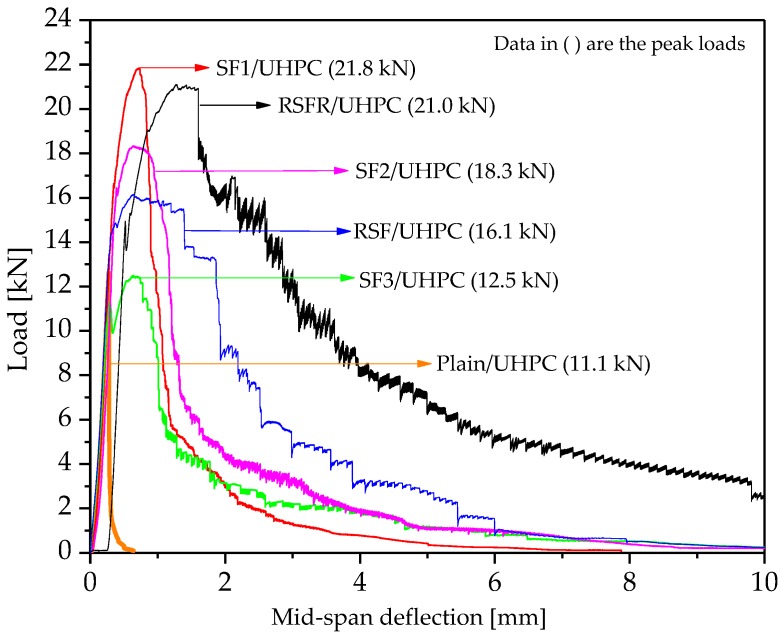
Load–deflection curves of various steel fiber-reinforced UHPC and plain UHPC during fracture energy testing.

Finally, the fracture energy of concrete was illustrated by its load–deflection curve during fracture energy testing, as shown in Figure 8. For UHPC reinforced with recycled steel fibers, after the peak load was reached, the load maintained almost no decrease. The descent stage of the curve was also much slower than that of UHPC with industrial steel fiber. These factors resulted in the fact that the load–deflection curve area of RSFR/UHPC was much larger than that of any other types of UHPC. During fracture energy testing, when cracking occurred, the concrete matrix lost its uncracked load-bearing capacity, and the steel fiber began to shoulder the load, displaying its post-peak loading toughness, which can be expressed by the descent stage of the load–deflection concrete curve. The peak load of the curve depends on the combined function between the load-bearing capacity of concrete and the post-peak loading toughness of steel fiber. Therefore, although the post-peak loading toughness of RSFR/UHPC was much better than industrial steel fiber, its uncracked load-bearing capacity was lower than that of UHPC with SF1. The negative effect of RSFR on the compressive strength of UHPC, as shown in Figure 3, demonstrates this fact. This effect led to the different trends between the peak load and the area of the load–deflection curve during fracture energy testing. 

**Figure 9:**
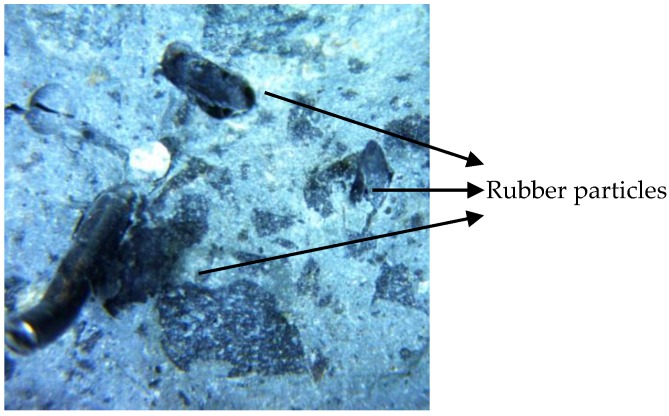
Rubber particles reserved in fiber holes on a fracture surface of a notched specimen after fracture energy testing.

RSFR displayed better post-peak loading toughness than RSF, which can be explained as follows. After cracking occurred during fracture energy testing, the rubber began shouldering the load (mainly tension) earlier than the steel fiber, and rubber’s high flexibility contributed to its tension resistance. Moreover, some rubbers displayed their preferable bonding strength with the concrete matrix. As shown in Figure 9, these rubbers remained in the matrix after the steel fiber was pulled out or ruptured. Conversely, the tensile strength of RSFR itself was much higher than that of RSF, which may be the most important factor. 

#### 3.1.4. Static Elastic Modulus 

**Figure 10:**
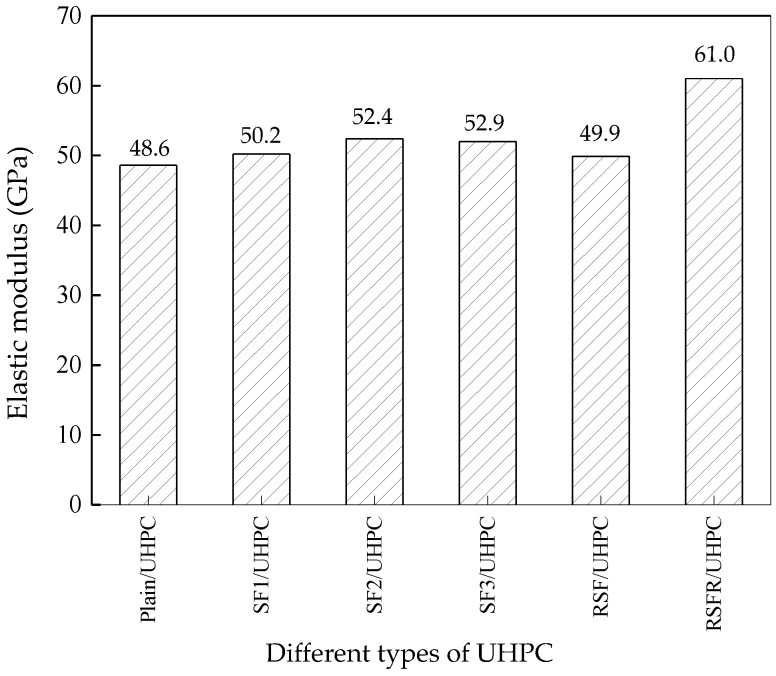
Elastic modulus of UHPC with various steel fibers and plain UHPC.

The static elastic modulus of UHPC is shown in Figure 10. It can be seen that RSFR displayed the most significant increase in the static elastic modulus of UHPC. The variation of the static elastic modulus of UHPC with various steel fibers was similar to that of the splitting tensile strength, except for RSF/UHPC. The static elastic modulus of RSF/UHPC was slightly lower than that of industrial steel fibers and much lower than that of RSFR/UHPC. RSF has a much lower stiffness than RSFR because of the damage it suffered when exposed to high temperatures of approximately 450 °C. The combined function of rubber and steel fiber with higher stiffness means that RSFR has a higher stiffness than RSF, although the attached rubbers decrease the stiffness of RSFR. 

### 3.2. Explosive Spalling Behavior

#### 3.2.1. Temperature–Time Curve during Explosive Spalling

**Figure 11:**
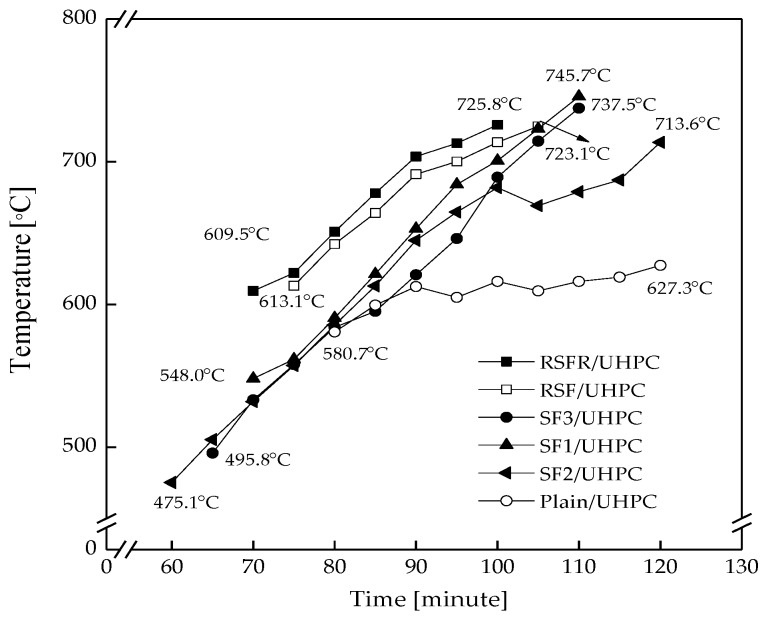
Temperature–time curves of all UHPC types during explosive spalling.

All types of UHPC specimens encountered explosive spalling in different temperature ranges, as presented in Figure 11. At temperatures lower than 400 °C, no spalling occurred for all types of specimens. After spalling occurred, different temperature ranges and durations of explosive spalling occurrence in different types of UHPC specimens could be observed, indicating that UHPCs with different types of steel fibers suffered significantly different severities of explosive spalling. SF2/UHPC suffered the most severe explosive spalling in the largest temperature range and the longest duration, followed by the SF3/UHPC. Recycled steel fibers significantly decreased the temperature range and the duration of the explosive spalling occurrence. There was an interesting result in which the temperature range of plain UHPC was the minimum, but its duration was not the shortest and the increase rate of temperature was the slowest. When explosive spalling occurred, moisture released from the spalled blocks and resulted in the decrease in temperature. The slower increase rate of temperature may imply the more serious explosive spalling. 

#### 3.2.2. Morphology of Spalled Specimens

**Figure 12:**
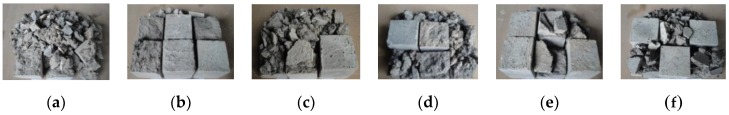
Morphology of UHSC with various steel fibers after subjected to a temperature of 800 °C. (**a**) Plain/UHPC; (**b**) SF1/UHPC; (**c**) SF2/UHPC; (**d**) SF3/UHPC; (**e**) RSF/UHPC; (**f**) RSFR/UHPC.

The morphology of all specimens after being subjected to 800 °C is shown in Figure 12. Plain UHPC suffered the severe explosive spalling, whereby six specimens spalled into fragments. Only one specimen remained intact without spalling occurrence for SF2/UHPC and SF3/UHPC. Based on the results in Figure 11, it can be concluded that the slower increase rate of temperature can be a parameter to estimate the severity of explosive spalling occurrence.

#### 3.2.3. Number of Spalled Specimens and Average Spalling Depth

The number of spalled specimens and the average spalling depth of six specimens of all concretes are given in Table 4. It indicates that all six specimens of the plain UHPC encountered explosive spalling. Adding steel fiber can relieve the explosive spalling severity of UHPC specimens, especially SF1 and recycled steel fibers, in which only three specimens spalled. To distinguish the spalling severity of SF1/UHPC, RSF/UHPC, and RSFR/UHPC, spalling depth of specimens was determined. The average spalling depth was the average value of the maximum spalling depth of six specimens. If no spalling occurred, the spalling depth of the specimen was confirmed as 0 mm. If the specimen spalled into several small fragments, its spalling depth was 100 mm. The average spalling depth of SF1/UHPC was only 12 mm, much smaller than other types of UHPC. SF1 exhibited the best influence on improving anti-spalling behavior.

#### 3.2.4. Particle Sieving

**Figure 13:**
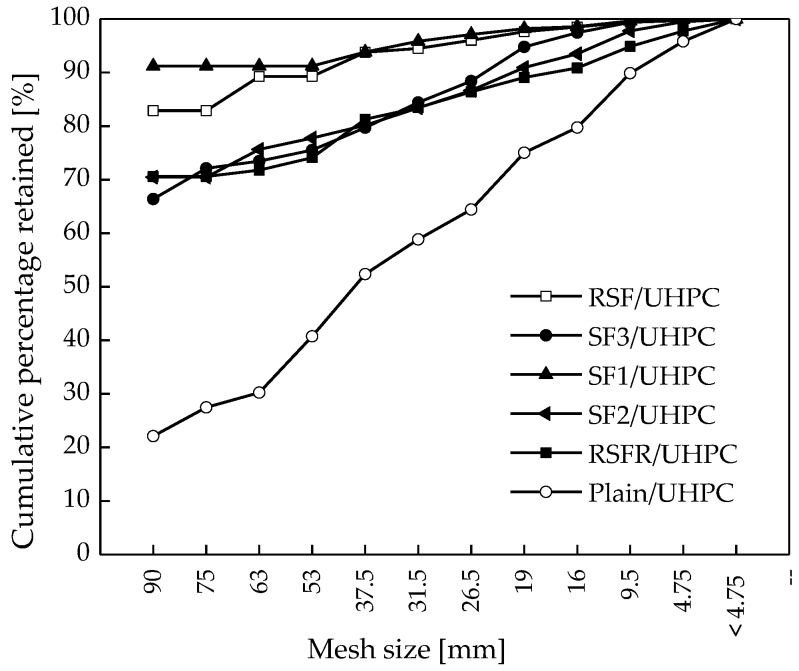
Particle size distribution of all types of specimens exposed to a temperature of 800 °C.

After the explosive spalling test, sieve tests were conducted on all of the UHPC specimens to further confirm the spalling degree of all concretes for comparison, as shown in Figure 13. For SF1/UHPC and RSF/UHPC, the mass percentages of particles with the sizes above 90 mm were 91% and 82%, respectively. In comparison, this percentage was only 20% for the plain UHPC. The results indicate that the explosive spalling of plain UHPC was extremely severe, as specimens spalled into many small fragments. Adding steel fiber can improve the anti-spalling behavior of UHPC.

#### 3.2.5. Analysis of Results

**Figure 14:**
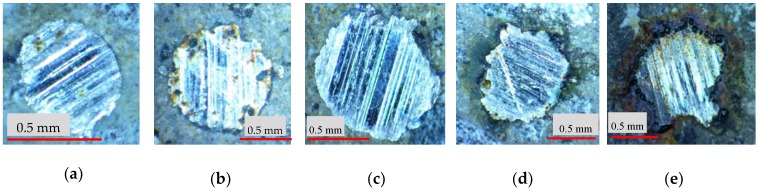
Observations of the interface zones between steel fibers and concrete matrix which specimens encountered no spalling by an optical microscope. (**a**) SF1; (**b**) SF2; (**c**) SF3; (**d**) RSF; (**e**) RSFR.

Based on the above results, it can be concluded that recycled steel fiber can improve the anti-spalling behavior of UHPC, and RSF had a better effect than RSFR. The beneficial effect of RSFR could be attributed to the rubber. After exposure to high temperature, melted rubber on the surface of RSFR created many channels, which released the vapor pressure, and thus impeded explosive spalling [59]. However, RSF unattached rubber showed a better effect than RSFR, and this result may be closely related to the fact that the content of the rubber attached to RSFR was approximately 10% of RSFR by mass. After exposure to high temperature, the attached rubbers with a high dosage can create much wider pores and cracks, which are detrimental to the anti-spalling behavior of concrete. For the RSF/UHPC, this adverse effect decreased due to the lower damage of the interface zone between RSF and concrete matrix compared to that of RSFR, as shown in Figure 14. 

Additionally, the above results indicate that recycled steel fibers significantly improved the fracture energy of UHPC. Explosive spalling is essentially severe cracking, and the anti-spalling behavior of concrete can be related to its fracture toughness with a positive relation. The result suggests that improvements of fracture toughness of concrete can be effective measures to enhance the resistance to explosive spalling of UHPC.

Compared to recycled steel fiber, industrial steel fiber SF1 presented the best effect on improving the anti-explosive spalling behavior of UHPC. This effect may be attributed to the higher distribution density of SF1. The number of SF1 and other types of steel fibers inside a 100 mm cube specimen were approximately 441 and 150, respectively. Steel fibers with a higher distribution density can significantly reduce the cracking of concrete. Additionally, SF1 can decrease thermal stress by improving the thermal conductivity property of concrete.

## 4. Discussion

The results in this experimental investigation indicate that the different types of steel fiber tested have a positive effect in improving the fracture energy and alleviating the explosive spalling severity of UHPC, but different efficiencies were presented. Industrial steel fibers SF1 with a smaller diameter have the most distribution density among all types of steel fiber, which could significantly decrease the inner temperature difference to reduce the thermal stress inside the specimens and caused the improvement of the anti-spalling behavior of concrete [60]. However, the smaller tensile strength of the SF1 limited its efficiency on improving the fracture energy of UHPC. Thus, compared to the improvement of steel fibers on the fracture energy of UHPC, the distribution density of steel fiber can be a more accurate parameter to evaluate its influence in improving the anti-spalling behavior of UHPC. However, the effect of inner thermal stress in inducing the explosive spalling occurrence of UHPC needs further research.

Recycled steel fibers not only improved the fracture energy of UHPC significantly but also alleviated the severity of explosive spalling occurrence in UHPC. This result indicates that the anti-spalling behavior of UHPC is closely related to the fracture toughness of concrete with a positive relationship, i.e., the higher the fracture toughness, the better the resistance to explosive spalling. UHPC with the industrial steel fibers SF2 and SF3 also have the same relationship trend between their fracture energies and the anti-spalling behaviors. Thus, to some extent, the steel fibers which can remarkably enhance the fracture energy of UHPC are able to improve the anti-spalling behavior of UHPC.

## 5. Conclusions

The results obtained from this work lead us to draw the following conclusions.

(1) Compared to plain ultra-high performance concrete (UHPC), adding industrial steel fiber and recycled steel fiber can increase mechanical properties of UHPC and alleviate its explosive spalling, except that recycled steel fibers with rubber particle attached (RSFR) have a slightly negative effect on the compressive strength of UHPC.

(2) Recycled steel fibers, especially RSFR, have the best effect on increasing the splitting tensile strength, fracture energy, and static elastic modulus of any industrial steel fiber. Compared to other properties in this experiment, the improvement of recycled steel fibers on the fracture energy of UHPC is the most significant. It is suggested that recycled steel fiber, especially RSFR, can be a toughening material used in concrete, and can substitute for industrial steel fiber in some concrete members, especially those with high toughness requirements. 

(3) All of the plain UHPC specimens encountered severe explosive spalling. Steel fiber significantly alleviates the explosive spalling of UHPC but cannot avoid the occurrence of explosive spalling. UHPC incorporating industrial hooked-end steel fiber (35 mm in length and 0.55 mm in diameter) exhibits the best resistance to explosive spalling. Recycled steel fiber, especially RSF, can also improve the anti-spalling behavior of UHPC. 

(4) Characteristics of steel fiber affect the mechanical properties, and explosive spalling behavior of steel fiber reinforced UHPC significantly. The steel fiber which has a large distribution density or that without a large distribution density but can make concrete have high toughness (fracture energy), is more appropriate to be utilized in UHPC structures. In addition, the application of recycled steel fiber is environmentally friendly and can help to efficiently save engineering costs.

## Figures and Tables

**Table 1:** Characteristics of steel fibers.

Type	Industrial Steel Fibers			Recycled Steel Fibers^1^	
	SF1	SF2	SF3	RSF	RSFR
Shape	Hooked-end	Hooked-end	Hooked-end	Corrugated	Corrugated
Nominal length (mm)	35	35	30	40	40
Nominal diameter (mm)	0.55	1	1	1	1.1
Aspect ratio	64	35	30	40	40
Tensile strength (MPa)	1100	900	1800–2000	>1250	1800–2000

^1^ Recycled steel fiber to which rubber was attached (RSFR), was treated by a high-temperature processing technique at approximately 450 °C to dispose of the rubber. Then, the recycled steel fiber with carbon black attached (RSF) was obtained and exhibited a lower tensile strength than RSFR.

**Table 2:** Mix proportions of ultra-high performance concrete (UHPC) (kg/m^3^).

Type	W/B	Binders		Artificial Sand	Coarse Aggregate	Steel Fiber		Super-Plasticizer
		C	SF*			Type	Content	
**Plain/UHPC**	**0.18**	**810**	**90**	620	930	—	0	7.2
SF1/UHPC	0.18	810	90	620	930	SF1	30	10.8
SF2/UHPC	0.18	810	90	620	930	SF2	30	10.8
SF3/UHPC	0.18	810	90	620	930	SF3	30	10.8
RSF/UHPC	0.18	810	90	620	930	RSF	30	12.6
RSFR/UHPC	0.18	810	90	620	930	RSFR	30	13.5

SF*, silica fume.

**Table 3:** Number of ruptured or pulled-out steel fibers on a surface of beam specimen during fracture energy testing.

Fiber	SF1	SF2	SF3	RSF	RSFR
Ruptured fiber	49	26	10	13	10
Pulled-out fiber	40	10	17	20	22

**Table 4:** Occurrence of explosive spalling of all UHSC types exposed to high temperature.

Type	Plain/UHPC	SF1/UHPC	SF2/UHPC	SF3/UHPC	RSF/UHPC	RSFR/UHPC
Number of spalled specimens	6(6)^1^	3(6)	5(6)	5(6)	3(6)	3(6)
Average spalling depth (mm)	92	12	82	76	27	36

^1^ The data inside the brackets indicate the amount of the total specimens for each condition. The data outside the brackets indicate the number of specimens that encountered explosive spalling.
